# Extreme Birth Weight and Metabolic Syndrome in Children

**DOI:** 10.3390/nu14010204

**Published:** 2022-01-02

**Authors:** Teofana Otilia Bizerea-Moga, Laura Pitulice, Cristina Loredana Pantea, Orsolya Olah, Otilia Marginean, Tudor Voicu Moga

**Affiliations:** 1 Department XI of Pediatrics—1st Pediatric Discipline, Center for Research on Growth and Developmental Disorders in Children, ‘Victor Babeș’ University of Medicine and Pharmacy Timișoara, Eftimie Murgu Sq no. 2, 300041 Timișoara, Romania; bizerea.teofana@umft.ro (T.O.B.-M.); marginean.otilia@umft.ro (O.M.); 2 1st Pediatric Clinic, ‘Louis Țurcanu’ Children’s Clinical and Emergency Hospital, Iosif Nemoianu 2, 300011 Timișoara, Romania; cristina.pantea13@yahoo.co.uk (C.L.P.); olah.orsi@yahoo.com (O.O.); 3 Department of Biology-Chemistry, West University of Timişoara, Pestallozi 16, 300115 Timişoara, Romania; 4 Laboratory of Advanced Researches in Environmental Protection, Oituz 4, 300086 Timişoara, Romania; 5 Department VIII of Neuroscience—Psychology Discipline, ‘Victor Babeș’ University of Medicine and Pharmacy Timișoara, Eftimie Murgu Sq no. 2, 300041 Timișoara, Romania; 6 Department VII of Internal Medicine—Gastroenterology Discipline, Advanced Regional Research Center in Gastroenterology and Hepatology, ‘Victor Babeș’ University of Medicine and Pharmacy Timișoara, Eftimie Murgu Sq no. 2, 300041 Timișoara, Romania; moga.tudor@umft.ro; 7 Gastroenterology and Hepatology Clinic, ‘Pius Brînzeu’ County Emergency Clinical Hospital, Liviu Rebreanu 156, 300723 Timișoara, Romania

**Keywords:** metabolic syndrome, extreme birth weight, gestational age, obesity, children

## Abstract

Small and large birth weights (BWs) for gestational age (GA) represent extremes, but the correlation between extreme BW and metabolic syndrome (MetS) has not been fully elucidated. In this study, we examined this correlation in obese children based on changes in their metabolic profile from childhood to adolescence. A retrospective observational study was performed on 535 obese patients aged 0–18 years in the Clinical and Emergency Hospital for Children “Louis Turcanu” in Timisoara, Romania, based on clinical and biological data from January 2015 to December 2019. We emphasized the links between extreme BW and obesity, extreme BW and cardiometabolic risk, obesity and cardiometabolic risk, and extreme BW, obesity and MetS. Children born large for gestational age (LGA) predominated over those born small for gestational age (SGA). Our findings showed that BW has an independent effect on triglycerides and insulin resistance, whereas obesity had a direct influence on hypertension, impaired glucose metabolism and hypertriglyceridemia. The influences of BW and obesity on the development of MetS and its components are difficult to separate; therefore, large prospective studies in normal-weight patients are needed.

## 1. Introduction

Extreme birth weight (BW), involving babies born small for gestational age (SGA) or large for gestational age (LGA), has been associated with an increased metabolic risk in adulthood. In the first two years of life, children born outside the normal BW range tend to return to their genetically determined growth trajectory. “Catch-up” and “catch-down” growth usually compensate for both restricted and excessive intrauterine fetal growth, respectively [1,2].

Rapid weight gain during the catch-up growth phase in SGA infants occurs due to an increase in fat mass at the expense of lean mass [3,4,5]. The “catch-up fat” phenomenon significantly increases the risk of childhood obesity in these children, thereby linking low BW to insulin resistance (IR), impaired glucose tolerance (IGT), type 2 diabetes, hypertension, and cardiovascular disease [3,6,7,8].

LGA infants who do not enter the catch-down phase have increased adiposity, a higher body mass index (BMI), and a higher risk of developing early obesity [9]. Furthermore, some evidence shows that LGA babies are more likely to be overweight regardless of catch-down growth [10]. Consequently, being born LGA represents a considerable cardiovascular and metabolic risk [11,12].

The prevalence of SGA and LGA births fluctuates significantly in developed countries: 4.6–15.3 and 5–20%, respectively. This variation for SGA births becomes more pronounced and prevalent in developing countries where it is estimated between 27 and 41.5% [13]. In contrast, the proportion of LGA newborns in developing countries tends to be lower (1–14.9%), compared to Nordic countries, where it is as high as 20% [14].

In a systematic review, Friend et al., concluded that the median prevalence of metabolic syndrome (MetS) in children was notably higher in overweight and obese children, with an occurrence of 11.9 and 29.2%, respectively. In contrast, in the whole population, the MetS criteria was met by 3.3% of cases [15]. Another review published by Reiner reported a range of MetS from 30 to 72% [16]. Two studies on the prevalence of MetS in obese children from Romania found it to be between 12.47 and 58% [17,18].

It was demonstrated that obese SGA children have an overall higher prevalence of MetS compared to those born appropriate for gestational age (AGA) (11 versus 9%), and it increases with age. This finding can be best observed in the adolescent group, where those born SGA had the highest prevalence of MetS (26.31%) compared to 16.84% of those born AGA [18]. The same tendency was described by Meas et al. in adults born SGA, who showed an increased risk of developing MetS compared to those born AGA. By the age of 30, 4.45% of AGA and 6.77% of SGA individuals met three MetS criteria; only 0.43% of AGA and 2.08% of SGA individuals met four MetS criteria [19].

Obese children born LGA are more prone to developing MetS, as observed in a study published by Wang et al., who found that the 65%prevalence of MetS in obese children born LGA, was significantly higher than for the 42.3% of obese children born AGA [20].

In this study, our aim was to examine the correlation between extreme BW and metabolic syndrome in overweight and obese children following changes in their metabolic profile from childhood to adolescence.

## 2. Materials and Methods

### 2.1. Definitions

The literature defines SGA as a BW and/or length less than −2.0 standard deviation (SD) or as a BW below the 10th percentile. LGA is defined as BW and/or length greater than +2.0 SD, and a BW greater than the 90th percentile [21,22,23,24]. Here, we used the definitions based on SD to determine the BW extremes [21,22,24].

Overweight and obesity are defined using age-specific BMI reference guidelines from the 2000 Centers for Disease Control and Prevention Child Growth Charts. Thus, overweight assumes BMI values between the 85th and 95th percentiles, and obesity implies values above the 95th percentile [25,26,27].

The following components of MetS were also defined: (1) obesity if BMI ≥ 95th percentile for age; (2) hypertension if systolic blood pressure ≥ 95th percentile for age; (3) impaired glucose metabolism if insulinemia ≥ 15 mU/L or fasting blood glucose ≥ 6.11 mmol/L; (4) dyslipidemia if triglyceride level ≥ 1.69 mmol/L or high-density lipoprotein (HDL) cholesterol < 0.91 mmol/L or total cholesterol ≥ 95th percentile. For the diagnosis of MetS, the presence of obesity plus at least two other components was required [28].

In this study, IR was evaluated by using the homeostasis model assessment for IR index (HOMA − IR), calculated using the following equation [29,30,31].

HOMA − IR = fasting insulin (μU/mL) × fasting plasma glucose (mmol/L)/22.5

IR was defined if the HOMA-IR exceeded the 95th percentile for age [29,30].

### 2.2. Study Design

This retrospective observational study was conducted in the Departments of Pediatric Endocrinology and Diabetes of “Louis Ţurcanu” Children’s Clinical and Emergency Hospital in Timișoara, Romania, over five years, from 1 January 2015 to 31 December 2019.

The study included 535 patients aged 0–18 who had been diagnosed with obesity. A retrospective analysis of clinical and biological data from the electronic register and patient files was performed. The patients were selected according to the following exclusion criteria: SGA/LGA of genetic or endocrine cause; pre- and post-maturity; monogenic or syndromic obesity; endocrine obesity (e.g., hypothyroidism, Cushing’s syndrome). Pre- and post-maturity have also been linked to early metabolic disturbances and therefore excluded from the current study in the attempt to reduce the number of variables influencing the cardio-metabolic risk.

The ethics committee of “Louis Ţurcanu” Children’s Clinical and Emergency Hospital in Timișoara approved the use of anonymized data sets of patients in the study without obtaining individual consent because only pre-existing clinical and biological data were interpreted, thus obviating the need for additional patient intervention.

**Figure 1:**
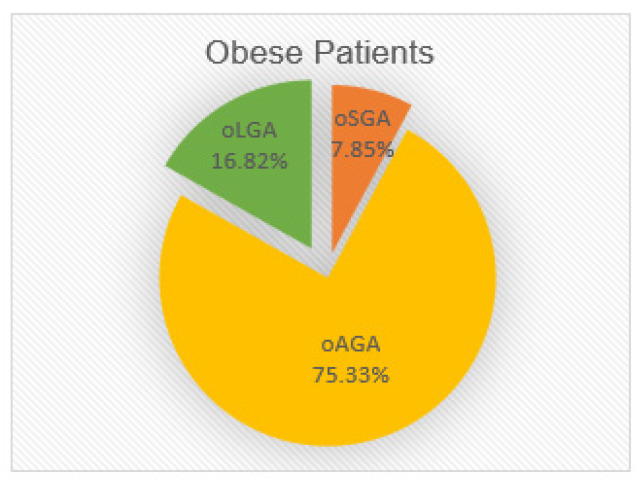
Percentage distribution of patients obese small for gestational age (oSGA), obese appropriate for gestational age (oAGA), and obese large for gestational age (oLGA) included in the study according to birth weight (BW) for gestational age (GA).

The 535 obese patients were divided into groups according to certain anthropometric parameters, height, and birth weight, relative to gestational age. Three study groups were formed: 42 obese SGA (oSGA), 403 obese AGA (oAGA), and 90 obese LGA (oLGA) subjects (Figure 1).

### 2.3. Measurements and Analytical Determinations

The determination of obesity in children was based on clinical evaluation and anthropometric measurements, as follows: actual weight (kg) and height (cm). Height was measured using an infant meter for children under 2 years of age and a fixed stadiometer for those 2 and over. Weight was determined using infant scales for children under 2 years of age and standing scales for those older. Based on these parameters, the BMI (kg/m^2^) was calculated using the formula: weight (kg) divided by height squared (m^2^) [32]. The BMI was then interpreted using BMI-for-age and -sex charts.

In all obese children in the study, BW was classified as SGA, AGA, or LGA after analyzing the patient’s files and applying the SD criteria for defining BW relative to GA [21,22,24].

A calibrated sphygmomanometer was used to determine systolic and diastolic blood pressure (BP), considering at least 2 values obtained at different times in a relaxed environment according to the recommendations of the National High Blood Pressure Education Program Working Group on High Blood Pressure in Children and Adolescents [33]. The obtained values were registered on corresponding percentiles. Arterial hypertension was diagnosed in children if the systolic BP were greater than, or equal to, the 95th percentile for age and sex.

Venous blood samples were taken in the morning, after a fasting period of at least 8 h to determine blood glucose, insulinemia, and lipid profile (total cholesterol, low-density lipoprotein (LDL cholesterol, HDL cholesterol, and triglycerides). The fasting glucose and lipid profiles were analyzed by colorimetric enzymatic spectrophotometry using the Cobas Integra 400 Plus^®^ analyzer from Roche Diagnostics, Rotkreuz, Switzerland. Fasting insulinemia was measured by electrochemiluminescence using the Cobas e411^®^ analyzer from Roche Diagnostics.

The standard oral glucose tolerance test (OGTT) was performed according to the recommendations of the International Society for Pediatric and Adolescent Diabetes (ISPAD) [34]. Fasting glucose levels were determined, followed by the oral administration of anhydrous glucose dissolved in water. The dose of oral glucose was adapted according to the patient’s weight: 1.75 g/kg body weight not exceeding 75 g. Glucose levels were measured again 2 h after oral glucose load.

### 2.4. Statistical Analysis

Version 26 of IBM SPSS (Statistical Product and Service Solutions, IBM, Ney York, NY, USA) software was used for the statistical processing of data. Continuous data without Gaussian distribution were described using median values (interval quartile) with the range of values presented. Nominal data are presented using absolute frequencies (percent). The Mann–Whitney U-test was used to compare the averages of the parameters among the groups. The state of normality of continuous variable distributions was tested using the Kolmogorov–Smirnov test, and the equality of variations was tested using Levene’s test. The regression and correlation analyses were performed to determine the degree of correlation, the causal relationships between the studied variables, and to determine whether the results were valid in terms of statistical significance. The degree of correlation (rho) between the parameters was assessed by calculating the Spearman correlation coefficient. A value of the statistical significance coefficient *p* < 0.05 was considered significant.

## 3. Results

A total of 535 obese children were examined to establish the link between BW and GA and MetS components. The median age of all obese children was 12.20 (9.60–14.90) years. The median gestational age of the subjects was 39 (38–40) weeks, and the median BW was 3300 (3000–3800) g with the oLGA group recording values almost double those of the oSGA group.

The percentage distribution of the 535 obese patients included in the study, according to BW for GA, is shown in Figure 1. The clinical and metabolic characteristics of the study groups divided according to BW for GA are presented in Table 1.

### 3.1. Extreme BW and Obesity

Figure 1 and Table 1 show that BW extremes for GA (SGA + LGA) represented about one-quarter of the total obese patients included in the study. It can also be seen that the percentage of obese SGA patients was about half of that of obese LGA patients. Regarding the relation between BMI and BW, the analysis of median values by study groups (Table 1) indicated a slight increase in the median BMI value in the oSGA and oLGA groups compared to the oAGA control group (29.03 and 29.12 vs. 28.21).

For all obese children, Table 2 shows the following correlations: BMI versus age, BMI versus BW, and BMI according to age and BW based on single and multiple regression analysis.

**Figure 2:**
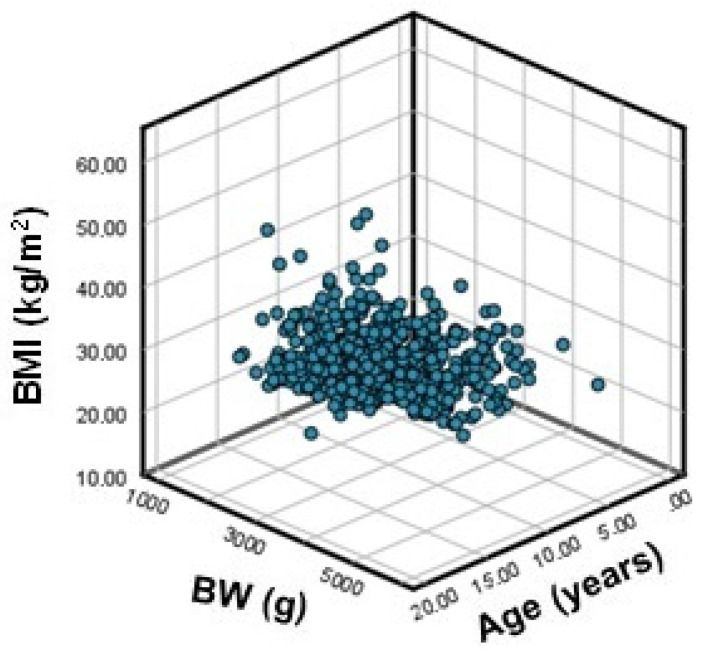
Correlation between BMI, age, and BW.

Figure 2 shows the correlations between BMI side versus age and BW.

Regarding the relationship between BMI and age, the coefficient of determination r^2^ = 0.297 (*p* < 0.001) showed the reduced effect of age on BMI: 29.7% of the BMI increase was determined by an increase in age. Regarding the relationship between BMI and BW, a simple linear regression analysis showed that no relationship was statistically significant (r^2^ = 0.005 for *p* = 0.094).

Multiple regression analysis (Figure 2) was applied to determine the variation in BMI according to age and BW, which revealed that 30.7% of the BMI increase in obese children (r^2^ = 0.307 for *p* < 0.001) was determined by the combined effect of increases of BW and age.

### 3.2. Extreme BW and Cardiometabolic Risk

**Figure 3:**
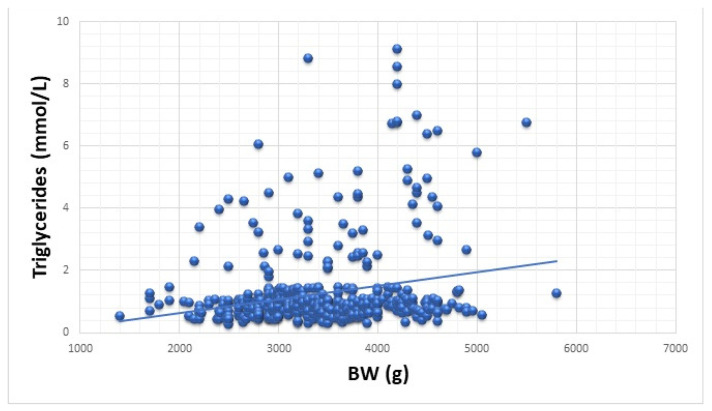
Correlation between BW and triglycerides.

Table 3 shows the linear regression correlations between BW and the individual MetS components (systolic and diastolic BP, fasting glucose, 2 h glucose, insulinemia, HOMA, cholesterol, HDL and LDL cholesterol, and triglycerides). The scatter plot depicted in Figure 3 shows the correlation between BW and triglycerides.

Considering the parameters that describe IR (Table 1), the most affected group was that of oSGA patients. Significantly higher values were recorded for insulinemia (11.48 vs. 9.05 and 9.13, respectively) and the HOMA index (2.68 vs. 1.79 and 1.84, respectively). However, linear regression analysis (Table 3) showed no statistically significant correlation between BW and insulinemia or BW and HOMA index (r^2^ = 0.002 and *p* = 0.339 for insulinemia and r^2^ = 0.004 and *p* = 0.168 for HOMA index).

Regarding dyslipidemia, although we observed no differences among the study groups in total cholesterol, lower values of HDL cholesterol were noticed among obese children born with extreme BW for GA (1.32 ± 0.44 in the oSGA and 1.47 ± 0.68 in the oLGA group) compared to obese individuals born AGA (2.08 ± 0.66), but with no statistical significance.

The slight increase in the median values from the oSGA to the oLGA group presented in Table 1, as well as the linear regression analysis (Table 3 and Figure 3), indicated a moderate effect of BW on triglycerides. It can be stated that a higher BW led to a higher level of triglycerides in these obese children (r^2^ = 0.050 and *p* < 0.001).

The other MetS components (systolic and diastolic BP, fasting and 2 h glucose) did not correlate with BW. This can be observed both from the median values presented in Table 1 and from the linear regression analysis in Table 3.

### 3.3. Obesity and Cardiometabolic Risk

**Figure 4:**
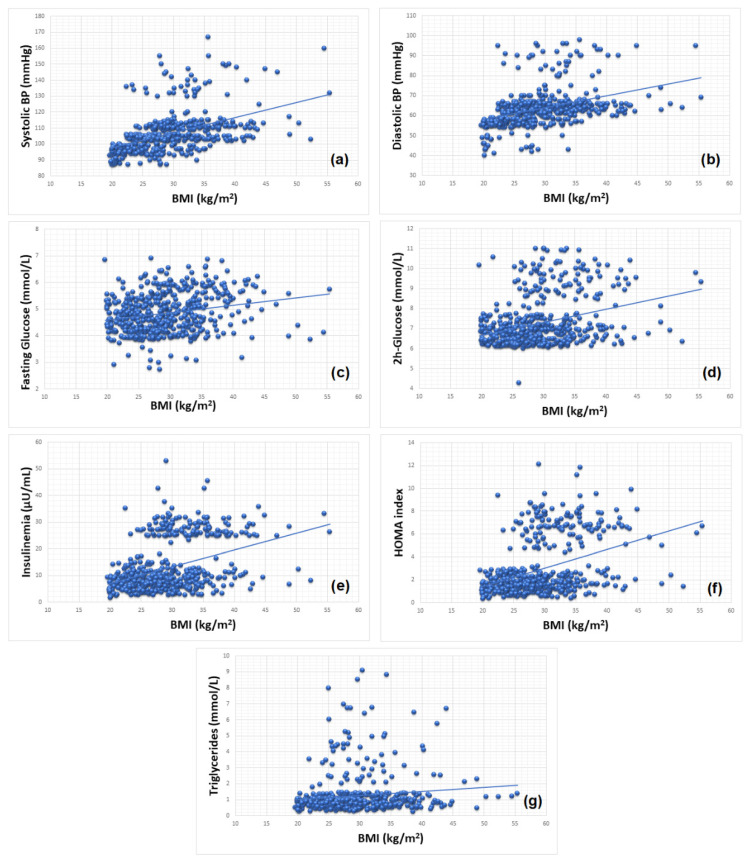
Correlation between BMI and (**a**) systolic blood pressure (BP), (**b**) diastolic BP, (**c**) fasting glucose, (**d**) 2 h glucose, (**e**) insulinemia, (**f**) HOMA index and (**g**) triglycerides.

Correlations using linear regression between BMI, and the individual MetS components (systolic and diastolic BP, fasting and 2 h glucose, insulinemia, HOMA, cholesterol, HDL and LDL cholesterol and triglycerides) are shown in Table 4 and Figure 4.

The statistical analysis results presented in Table 4 show that between BMI and systolic and diastolic BP (Figure 4a,b), fasting and 2 h glucose (Figure 4c,d), insulinemia and HOMA index (Figure 4e,f), and triglycerides (Figure 4g), a statistically significant, positive, low-intensity correlation exists. Statistical analysis also showed that the relationship between BMI and total cholesterol level (r^2^ = 0.005 at *p* = 0.097), HDL cholesterol (r^2^ = 0.001 at *p* = 0.730). and LDL cholesterol (r^2^ = 0.001 at *p* = 0.441) was not statistically significant (Table 4).

### 3.4. Extreme BW, Obesity and MetS

We also examined the clustering of MetS components (hypertension, hyperinsulinemia, impaired fasting glucose or glucose tolerance, hypercholesterolemia, hypertriglyceridemia, and low HDL cholesterol) in the obese children included in the study groups. The aim was to determine and compare the prevalence of MetS among these factors.

Table 5 shows the distribution of the MetS components in the studied groups of children with obesity.

As can be seen in Table 5, among the 535 patients included in the study, in addition to obesity, hyperinsulinemia (137, 25.60%) and impaired glucose tolerance (105, 19.63%) predominated as the two criteria that described carbohydrate metabolism impairment as a component of MetS. However, we noted that fasting blood glucose was not an equally accurate parameter reflecting glucose metabolism disorders.

The highest number of children with dyslipidemia, as defined by high cholesterol and triglycerides, and low HDL cholesterol can be observed in the oLGA group (16.67%, children with hypercholesterolemia; 25.56%, with hypertriglyceridemia; 14.44%, with low HDL cholesterol), followed by the oSGA group (9.52% of patients with hypercholesterolemia, 11.90% with hypertriglyceridemia, and 7.14% with low HDL cholesterol) compared to the AGA control group (5.71% of children with hypercholesterolemia, 9.43% with hypertriglyceridemia, and 2.98% with low HDL cholesterol).

**Figure 5:**
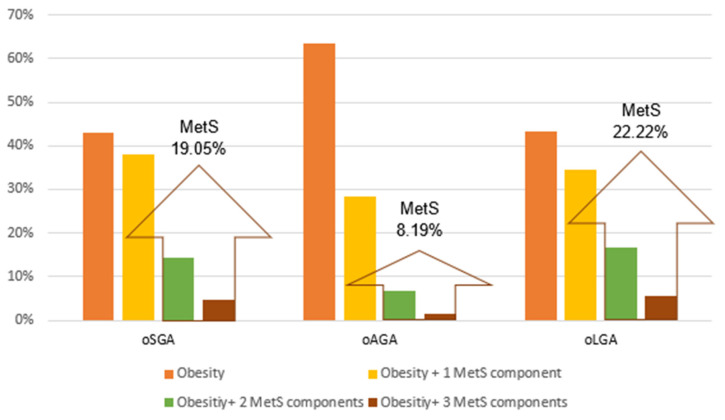
The percentage distribution of metabolic syndrome (MetS) and components among the study groups.

Figure 5 shows the percentage distribution of MetS and its components in the groups. According to the criteria of Viner et al., which were applied in the study, MetS is defined if obesity and at least two other components (hypertension, impaired glucose metabolism, and dyslipidemia) are present in the same person [28].

## 4. Discussion

### 4.1. Extreme BW and Obesity

The prevalence of extreme BW related to GA mentioned the in literature is wide-ranging. Taal et al. [9], in a study of 3941 children of normal weight, found no significant percentage differences between the BW extremes (191 SGA subjects representing 4.84% and 199 LGA subjects accounted for 5.04% of the total). Two other prospective studies conducted on a normal-weight population both found SGA prevalence to be 26.6%, but the LGA prevalence varied significantly. Chiavaroli et al. [35] recorded 34.4% LGA patients (31 of 90 total patients), representing a higher percentage compared to the SGA group, whereas Lurbe et al. [36] discovered a much lower percentage (14.4%) in the LGA patients. Another study conducted between 1993 and 2013 on a pooled total of 5896 live births did not find significant differences regarding the incidence of SGA or LGA births twenty years apart (8.3 and 10.8% in 1993 versus 7.6 and 11.7% in 2013, respectively) [13]. In the current study, the prevalence of SGA was 7.85% and that for LGA was 16.82%. The same bias in favor of LGA (20% LGA compared to 7% SGA) was described by Hill et al. [37] in their study, which was also conducted on obese patients.

The slightly increased median BMI value in the oLGA group can be explained by the finding that LGA-born children who did not “catch down” maintained their excess weight throughout growth and development. This observation was reinforced by the finding that the combined effect of increases in BW and age led to a BMI increase in 30.7% of study subjects. This finding was in agreement with other research findings [9,11,12,14,38,39]. In their study, Hill et al., found a positive correlation between BMI and BW for GA in adolescents diagnosed with obesity [37]. A follow-up study performed by Lurbe et al. on 139 children at 5 years of age found that out of the 27% of subjects that developed obesity, 50% were born LGA, 25% SGA and 18% AGA [36]. Other authors claimed that both BW extremes for GA were linked to an increased risk of obesity later in life. Although a correlation between LGA and persistent overweight throughout childhood and adolescence seems more logical, weight catch up in SGA-born children in the first years of life can also lead to excessive adipose tissue deposition and visceral adiposity [38]. In LGA-born children, overweight and obesity appear at a younger age compared to SGA-born children [24]. The contradictory research results as well as the finding that only one-quarter of patients’ BMI values were influenced by BW and age combined to indicate the need to extend research by conducting a prospective study to follow normal-weight children from infancy to adolescence, following both the presence of MetS components and lifestyle habits.

### 4.2. Extreme BW and Cardiometabolic Risk

Scientific evidence for the link between birth weight and blood pressure is controversial. Additionally, no consensus exists regarding the major determinant of hypertension, extreme BW, or subsequent weight gain. Some authors have stated that SGA is associated with elevated systolic BP [6,19,24,40], whereas others have claimed that LGA is strongly linked to high BP and increased cardiovascular risk [20,24,41]. In our study, we did not find any correlation between BW and systolic or diastolic BP, which is consistent with the observations of other researchers who found no link between BW and hypertension [35,37]. Some prospective studies conducted on lean children and adults demonstrated a connection between weight gain rather than BW and high BP, respectively [36,42].

No detectable variations were found in the current study regarding the median fasting glucose and glucose levels at 2 h of an OGTT, between oSGA, oAGA, and oLGA groups. Other studies found differences between the plasma glucose concentration at 120 min during an OGTT of SGA-born individuals compared to those born AGA [19,40], whereas Xiao et al. found overall impaired glucose metabolism in SGA children [43]. A higher, but not large, BW appears to offer protection against glucose intolerance [44].

In this study, fasting insulinemia and the HOMA index describing IR had non-statistically significant higher median values among oSGA children compared to both oAGA controls and oLGA subjects, a finding that was consistent with the literature. Researchers agreed that SGA had a strong influence on later IR development [6,19,35,36,37,40]. However, some studies also reported a stronger correlation between LGA and IR compared to AGA [24,35,45,46]. Our data did not show a higher IR in oLGA children compared to oAGA controls, as was also found by Huang et al. [47].

Whereas IR is associated with SGA, hypertriglyceridemia is associated with LGA. Three hypotheses are used to explain the development of IR in obese SGA children. The “thrifty phenotype” hypothesis refers to metabolic reprogramming caused by malnutrition in the embryo–fetal period. Under conditions of normal or excessive nutrition after birth, the children develop insulin resistance later in life. The “fetal salvage” postulation states that IR in an undernourished fetus develops to redistribute essential nutrients like glucose to vital organs, mainly the brain. Finally, the “catch-up growth” theory considers IR as a consequence of a sudden exposure to high concentrations of insulin and insulin-like growth factors in the postnatal period as a response to adequate or overnutrition, after intrauterine undernutrition and depletion of the two hormones [48].

The lipid profile did not show differences between the median values of total cholesterol in the groups. Lower values of HDL cholesterol were found among obese children born with extreme BW for GA compared to obese born AGA, but with no statistical significance. Whereas some studies did not find any detectable differences in the lipid profile of SGA, AGA, or LGA children [35], others reported an inverse association between total cholesterol and BW [49]. Meas et al. found no differences between SGA and AGA children regarding total plasma cholesterol, but a significantly decreased plasma HDL cholesterol concentration was found in the SGA group compared to the AGA controls [19]. The same observation was reported by Lurbe et al., when comparing both AGA and LGA children to their SGA peers [36]. Extreme BW for GA in adults has been associated with dyslipidemia [50].

Current research data has shown that BW exerts a moderately positive effect on triglyceride values, with statistical significance. Thus, a higher BW will lead to higher triglyceride values. Other authors reached the same conclusion [36], whereas some reported a high prevalence of hypertriglyceridemia in SGA compared to AGA children [51].

Although the literature regarding the pathophysiological mechanism of hypertriglyceridemia in LGA children is scarce, this link is highlighted by many studies looking at the relationship between LGA, obesity, and MetS. We think that this relationship deserves further research as hypertriglyceridemia is an important biomarker of cardiometabolic risk in these children.

### 4.3. Obesity and Cardiometabolic Risk

Concerning the relationship among BMI, MetS, and its components, we found a statistically significant positive correlation among BP, fasting and 2 h glucose, IR (insulinemia and HOMA index) and triglycerides, but no correlation was observed regarding total cholesterol, and HDL and LDL cholesterol. Two studies conducted on Argentinean and Saudi children concerning the relationship between obesity and MetS reported similar findings [52,53].

The mechanism through which obesity causes hypertension in children is not fully understood. A combination of mutually potentiating factors have been considered, including increased activity of the sympathetic nervous system and the renin–angiotensin–aldosterone axis, excessive secretory function of adipose tissue, insulin resistance, and vascular remodeling [54,55,56,57].

Decreases in glucose tolerance and insulin sensitivity accompany one another. As an important marker of cardiometabolic risk, IR is linked to both obesity and SGA. However, obesity plays the most important role in its development.

Triglycerides, another biomarker of cardio-metabolic risk, show an important positive correlation with both BW and current weight. In obese children, hypertriglyceridemia is determined by an impaired storage of triglycerides in the adipocytes and release of fatty acids [58].

### 4.4. Extreme BW, Obesity and MetS

In addition to obesity, the MetS components most prevalent in the children were hyperinsulinemia and impaired glucose tolerance. An important obesity-related imbalance is that of the carbohydrate metabolism, as ascertained by our research as well as several other studies [59,60,61,62,63]. Although many authors proposed fasting glucose as a reliable parameter to describe impaired glucose tolerance, when defining the MetS components [60,61,63], in our study, glucose levels at two hours of an OGTT proved to be a more accurate marker in this regard. Other researchers, such as Viner et al. and Weiss et al., arrived at the same conclusion [28,62].

Dyslipidemia was predominant in oLGA children, where the highest prevalence of high total cholesterol, triglycerides, and low HDL cholesterol were detected.

The prevalence of MetS in the obese children in this study was higher among those at both BW extremes for GA. Whereas some authors reported an increased influence of SGA on the development of MetS in childhood and adolescence [6,19,64,65,66], others found that this influence to be more pronounced in LGA [64,67,68].

### 4.5. Limitations of the Study

It is recognized that retrospective studies are affected by several limitations, which are also found in our study. The retrospective design implies that patient data has been collected from medical records and might therefore be more prone to selection and classification bias. A significant number of patients could not be included in the analysis due to missing or incomplete data. Factors interfering with postnatal weight gain and growth, like the timing of weaning and diet quality, were not recorded and therefore could not be considered. The lack of information might have influenced the direct link between small and large BW for GA and the prevalence of MetS. There was also limited information regarding patient family history, such as family body shape, dietary habits and illness. Both SGA and LGA groups were significantly smaller compared to the AGA control group; therefore, the study’s findings might not be representative of the general population. In addition, certain risk factors may have been overrepresented in the hospitalized subjects in the study compared with the general population.

## 5. Conclusions

The large variation in the prevalence of extreme BW for GA may have been influenced by the study premises. LGA is expected to predominate over SGA in an obese population. Although both extremes of BW are genetically programmed to return to a normal weight curve in the first years of life, LGA individuals are more likely to maintain weight excess from birth throughout growth and development. In normal-weight subjects, large differences in the prevalence of an extreme BW may be explained by other factors such as socio-environmental context, dietary habits and lifestyle.

Our study showed that BW has an independent effect on triglycerides and insulin resistance, which are known biomarkers of cardiometabolic risk. Although various theories have been developed to explain the link between IR and SGA, the association between BW and hypertriglyceridemia remains unclear. For this reason, we consider it necessary to intensify research in this area.

Obesity is known to be strongly linked to metabolic and cardiovascular disturbances. As such, our statistical analysis was expected to show a significant correlation between obesity and hypertension, impaired glucose metabolism, and hypertriglyceridemia. To evaluate glucose tolerance, we found that 2 h glucose of an OGTT was a more accurate marker.

Metabolic reprogramming followed by catch-up growth in SGA children will ultimately lead to obesity. The lack of catch down in LGA children is also strongly connected to obesity. Therefore, it was hard to separate the influences of BW and obesity on the development of MetS and its components. Prospective studies performed on large cohorts of normoponderal patients are required to observe the separate influences of BW.

The prevalence of MetS in obese SGA and LGA children was higher compared to AGA controls. Therefore, we can state that children born at both extremes of BW should be closely monitored.

## Figures and Tables

**Table 1:** Clinical and biological features of the study groups according to birth weight (BW) for gestational age (GA).

Study Group	oSGA		oAGA		oLGA	
Number of Children	42		403		90	
	Median	Range	Median	Range	Median	Range
Clinical Parameters						
Age (years)	13.20	3.40–18.11	12.40	0.50–19.90	11.85	0.11–18.90
Gestational age (weeks)	38	37–40	39	37–42	40	37–42
Birth weight (g)	2300	1400–2500	3250	2600–4000	4400	4050–5800
BMI (kg/m^2^)	29.03	19.63–38.53	28.21	19.84–55.38	29.12	19.98–44.92
Systolic blood pressure (mmHg)	105	93–167	104	87–160	103	88–148
Diastolic blood pressure (mmHg)	64	54–98	63	40–96	64	45–96
Metabolic Parameters						
OGTT: Fasting glucose (mmol/L)	4.96	3.94–6.85	4.75	2.73–6.92	4.83	3.88–6.87
OGTT: 2h-glucose (mmol/L)	7.03	4.28–11.01	6.86	6.01–11.02	6.76	6.05–11.02
Insulinemia (μUI/mL)	11.48	3.20–52.95	9.05	2.40–33.2	9.13	1.70–35.80
HOMA index	2.68	0.75–12.12	1.79	0.40–9.52	1.84	0.33–9.91
Total cholesterol (mmol/L)	3.89	2.75–9.35	4.21	2.49–9.20	4.20	2.44–8.77
HDL-cholesterol (mmol/L)	1.17	0.25–2.83	2.08	0.32–3.22	1.17	0.31–3.10
Triglycerides (mmol/L)	0.73	0.24–4.27	0.88	0.28–8.82	0.99	0.33–9.09

Abbreviations: oSGA = obese small for gestational age; oAGA= obese appropriate for gestational age; oLGA = obese large for gestational age; BMI = body mass index; OGTT = oral glucose tolerance test; HOMA = homeostasis model assessment; HDL = high-density lipoprotein.

**Table 2:** BMI regression analysis.

	r^2^	*p*	Slope Intercept
BMI vs. age (years)	0.297	<0.001	y = 19.161 + 0.841x
BMI vs. BW (g)	0.005	0.094	y = 27.01 + 0.001x
BMI vs. age (years) and BW (g)	0.307	<0.001	y = 16.007 + 0.001x + 0.848z

**Table 3:** Regression analysis versus BW.

	r^2^	*p*	Slope Intercept
Systolic blood pressure (mmHg)	0.003	0.190	y = 110.06 − 0.001x
Diastolic blood pressure (mmHg	0.001	0.638	y = 64.494 − 0.001x
OGTT: Fasting glucose (mmol/L)	0.004	0.146	y = 4.595 + 7.146x
OGTT: 2 h glucose (mmol/L)	0.002	0.330	y = 6.984 + 7.857x
Insulinemia (μUI/mL)	0.002	0.339	y = 10.845 + 0.001x
HOMA index	0.004	0.168	y = 2.145 + 0.001x
Total cholesterol (mmol/L)	0.001	0.515	y = 4.107 + 0.004x
HDL cholesterol (mmol/L)	0.005	0.105	y = 2.174 − 7.579x
LDL cholesterol (mmol/L)	0.007	0.058	y = 1.124 + 0.001x
Triglycerides (mmol/L)	0.050	<0.001	y = −0.236 + 0.001x

Abbreviations: LDL = low-density lipoprotein.

**Table 4:** Regression analysis versus BMI.

	r^2^	*p*	Slope Intercept
Systolic blood pressure (mmHg)	0.213	<0.001	y = 78.959 + 0.941x
Diastolic blood pressure (mmHg)	0.148	<0.001	y = 46.286 + 0.590x
OGTT: Fasting glucose (mmol/L)	0.049	<0.001	y = 4.029 + 0.028x
OGTT: 2 h glucose (mmol/L)	0.103	<0.001	y = 5.330 + 0.066x
Insulinemia (μUI/mL)	0.156	<0.001	y = −5.403 + 0.625x
HOMA index	0.158	<0.001	y = −1.841 + 0.162x
Total cholesterol (mmol/L)	0.005	0.097	y = 3.893 + 0.012x
HDL cholesterol (mmol/L)	0.001	0.730	y = 1.864 + 0.001x
LDL cholesterol (mmol/L)	0.001	0.441	y = 1.455 + 0.007x
Triglycerides (mmol/L)	0.014	0.005	y = 0.491 + 0.026x

**Table 5:** Distribution of the individual MetS components in the study groups.

Study Groups Parameters	oSGA		oAGA		oLGA		Total	
Number of Children	42		403		90		535	
	No	%	No	%	No	%	No	%
Hypertension	6	14.29	27	6.70	10	11.11	43	8.04
Impaired glucose metabolism								
Hyperinsulinemia	16	38.10	95	23.57	26	28.89	137	25.60
Impaired fasting glucose	3	7.14	14	3.47	7	7.78	24	4.49
Impaired glucose tolerance	14	33.33	70	17.37	21	23.33	105	19.63
Impaired lipid metabolism								
Hypercholesterolemia	4	9.52	23	5.71	15	16.67	42	7.85
Hypertriglyceridemia	5	11.90	38	9.43	23	25.56	66	12.34
Low HDL cholesterol	3	7.14	12	2.98	13	14.44	28	5.23

## Data Availability

The data are not publicly available due to reasons of privacy.
